# Complex nonlinear autonomic nervous system modulation link cardiac autonomic neuropathy and peripheral vascular disease

**DOI:** 10.3389/fphys.2015.00101

**Published:** 2015-03-27

**Authors:** Kinda Khalaf, Herbert F. Jelinek, Caroline Robinson, David J. Cornforth, Mika P. Tarvainen, Hayder Al-Aubaidy

**Affiliations:** ^1^Department of Biomedical Engineering, Khalifa University of Science, Technology and ResearchAbu Dhabi, UAE; ^2^Australian School of Advanced Medicine, Macquarie UniversitySydney, NSW, Australia; ^3^Centre for Research in Complex Systems and School of Community Health, Charles Sturt UniversityAlbury, NSW, Australia; ^4^School of Community Health, Charles Sturt UniversityAlbury, NSW, Australia; ^5^School of Design, Communication and Information Technology, University of NewcastleNewcastle, NSW, Australia; ^6^Department of Applied Physics, University of Eastern FinlandKuopio, Finland; ^7^Department of Clinical Physiology and Nuclear Medicine, Kuopio University HospitalKuopio, Finland; ^8^School of Medicine, University of TasmaniaHobart, TAS, Australia

**Keywords:** network physiology, complex systems, ankle brachial pressure index (ABPI), peripheral vascular disease (PVD), heart rate variability (HRV), calcification, atherosclerosis

## Abstract

**Background:**

Physiological interactions are abundant within, and between, body systems. These interactions may evolve into discrete states during pathophysiological processes resulting from common mechanisms. An association between arterial stenosis, identified by low ankle-brachial pressure index (ABPI) and cardiovascular disease (CVD) as been reported. Whether an association between vascular calcification—characterized by high ABPI and a different pathophysiology—is similarly associated with CVD, has not been established. The current study aims to investigate the association between ABPI, and cardiac rhythm, as an indicator of cardiovascular health and functionality, utilizing heart rate variability (HRV).

**Methods and Results:**

Two hundred and thirty six patients underwent ABPI assessment. Standard time and frequency domain, and non-linear HRV measures were determined from 5-min electrocardiogram. ABPI data were divided into normal (*n* = 101), low (*n* = 67) and high (*n* = 66) and compared to HRV measures.(DFAα_1_ and SampEn were significantly different between the low ABPI, high ABPI and control groups (*p* < 0.05).

**Conclusion:**

A possible coupling between arterial stenosis and vascular calcification with decreased and increased HRV respectively was observed. Our results suggest a model for interpreting the relationship between vascular pathophysiology and cardiac rhythm. The cardiovascular system may be viewed as a complex system comprising a number of interacting subsystems. These cardiac and vascular subsystems/networks may be coupled and undergo transitions in response to internal or external perturbations. From a clinical perspective, the significantly increased sample entropy compared to the normal ABPI group and the decreased and increased complex correlation properties measured by DFA for the low and high ABPI groups respectively, may be useful indicators that a more holistic treatment approach in line with this more complex clinical picture is required.

## Introduction

**Figure 1:**
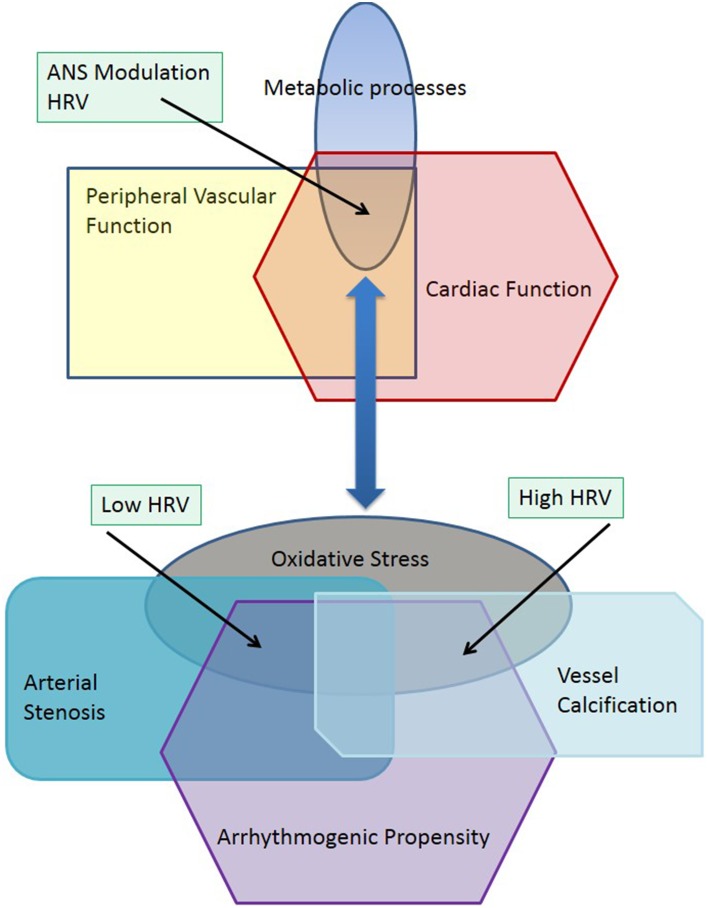
**Progression and divergence of peripheral vascular disease and HRV**.

The evolution of complex systems may be characterized by nonequilibrium phase transitions that lead to a divergence of structure and function in biological systems (Haken, 1983). Pathophysiological processes are multifactorial and characterized by chaotic dynamics where nonequilibrium phase transitions may lead to new multiple stable states including diabetes, chronic kidney disease or coronary artery disease, as well as peripheral vascular disease (Glass, 2001). Physiological or pathophysiological stable states may also include transient processes occurring in neuronal, metabolic or vascular function such as can be observed during the stages of sleep (Bashan et al., 2012). Peripheral vascular disease (PVD) is characterized by changes in structural and metabolic function in peripheral arteries and arterioles, and is generally considered to be a predictor of cardiovascular disease (CVD) and stroke (Thom et al., 2006). The ankle-brachial pressure index (ABPI) provides a tool for identification of patients at higher risk of adverse cardiovascular events (Hirsch et al., 2005). ABPI values range from 0.5 to above 1.4 indicating either arterial stenosis or vascular calcification (Jelinek and Austin, 2006). This divergent pathophysiology hinges on changes in metabolic processes such as oxidative stress (Loffredo et al., 2007), where increased reactive oxygen species (ROS) have been shown to have a causative role in atherosclerosis and vessel narrowing, thus leading to a lower ABPI (Dröge, 2002; Madamanchi et al., 2005). Different but overlapping metabolic processes are involved in vessel calcification (Demer and Tintut, 2008; Rogers and Aikawa, 2014). These subtle differences in metabolic processes affecting blood constituents and the vessel walls can lead to divergent stable states manifesting as arterial stenosis, which is due to a narrowing of the arterial lumen, or vascular calcification, which is characterized by arterial sclerosis. The autonomic nervous system, which, regulates heart rate and vessel diameter, is also affected by ROS through central or peripheral mechanisms (Feldman, 2003; Ye et al., 2006). These neural processes may then be coupled to different pathological stable states such as arterial stenosis and vascular calcification (Figure 1). Figure 1 explores the effect of pathophysiological changes to a common metabolic pathway regulating vascular and neural function such as oxidative stress through increases in reactive oxygen species (Feldman, 2003). When the physiological systems and metabolic processes are in equilibrium, the interaction of diverse physiological subsystems provide a stable functioning environment including interactions between the peripheral vasculature and cardiac rhythm (Madamanchi et al., 2005). This is depicted in the top part of the figure. Oxidative stress is a manifestation of a changed metabolic milieu, which in turn leads to divergent pathophysiological functions as depicted in the lower half of the diagram illustrated as arterial stenosis, vessel calcification and arrhythmogenic propensity. The physiological coupling between vascular and cardiac function has changed and can be seen in a similar way that a phase transition leads to new subsystems (Haken, 1983). Arterial stenosis and vascular calcification can be viewed as two new subsystems, which are now differently coupled to cardiac rhythm. That is arterial stenosis and low ABPI is now associated with a reduced heart rate variability (HRV) and vessel calcification is associated with increased HRV. Both increased and decreased HRV, which may be associated with a decrease or increase in the time and frequency domain HRV features or indicated by lower or higher entropy as well as changes in short and long-term correlations between RR intervals measured by DFA. Increased and decreased HRV have been shown to increase the risk of adverse cardiac events (Stein et al., 2005).

Individual divergent states are coupled to produce cooperative states with other physiological systems including the cardiovascular and renal systems. Investigating both the ABPI—a measure of peripheral vasculature patency and vessel wall stiffness, and heart rate variability (HRV)—analysis of cardiac rhythm, (Goernig et al., 2008; Deguchi et al., 2009; Jelinek et al., 2013) may provide insight into the relationship of vascular networks and their coupling with the autonomic nervous system.

HRV analysis is a simple, sensitive and non-invasive method for measuring cardiac rhythm and refers to the beat-to-beat variation in heart rate. It is the result of complex interactions between the autonomic nervous system, endocrine influences, vasomotor and respiratory centers (Kautzner and Camm, 1997; Chandra et al., 2003). An increase or decrease in HRV typically provides an indication of compromised cardiovascular health and increased risk of cardiac morbidity and mortality (Task Force, 1996; Pumprla et al., 2002).

In clinical practice, peripheral vascular function is most often determined using the ankle brachial pressure index (ABPI). ABPI measures the patency of blood vessels in the lower limbs. A lower ABPI usually indicates arterial stenosis due to the formation of atheromatous plaques in the vessel walls, whereas an elevated ABPI indicates arterial sclerosis due to a more pronounced calcification of the vessel wall. Based on the literature, normal values for ABPI, independent of age and gender, should be above 0.9 and below 1.3 (Rose, 2000; Brooks et al., 2001; McDermott McGrae et al., 2003b). Results below this threshold indicate varying levels of peripheral arterial stenosis. An ABPI over 1.3 indicates vessel wall calcification. However, consensus on cut-off values for low or high ABPI associated with pathology has not been reached, and the normal range of ABPI values used in clinical practice may range between 0.8 and 1.4 (Hiatt et al., 1995; Moffatt and O'Hare, 1995; Rose, 2000; Brooks et al., 2001; Vowden and Vowden, 2001; McDermott McGrae et al., 2003a; Resnick et al., 2004; McDermott et al., 2005; Austin et al., 2006; Jelinek and Austin, 2006). The prevalence of low ABPI in clinical practice typically varies with age and presence of cardiovascular risk factors, ranging from 2 to 29%. Coronary artery disease is present in approximately half of the individuals presenting with a low ankle brachial pressure index (ABPI), based on ECG findings (Hirsch et al., 2006). Conversely, the prevalence of vascular calcification, which is more commonly associated with increased ABPI values, has been reported to be between 1.1 and 6.3% depending on age and the ABPI cut-off value chosen (Wattanakit et al., 2007; Allison et al., 2008). The pathophysiological relationship between CVD and high ABPI remains unknown, although many studies have addressed the correlation between low ABPI and CVD (Cui et al., 2014; Zhan et al., 2015). This may be in part due to the two different, complex dynamic processes associated with the development of arterial stenosis and vascular calcification.

It is well established that CVD risk increases with decreased or increased ABPI. In addition, CVD or coronary heart disease mortality has been shown to be associated with abnormal heart rate variability (HRV) (Huikuri, 1995; Voss, 2007; Jelinek et al., 2013; Papaioannou et al., 2013).

Up to now no laboratory has investigated the changes in HRV associated with both low ABPI (arterial stenosis) and high ABPI (vascular calcification) and the effect on HRV. How pathological changes to the metabolic network lead to different expressions within the vascular networks, and how in turn these are coupled with the cardiac network, requires further investigation. Specifically, we address the metabolic/pathophysiological coupling associated with arterial stenosis and vascular calcification with respect to ABPI and HRV measures in our model.

## Methods

### Participant recruitment

Participants were recruited as part of the Charles Sturt University Allied Health Clinic diabetes complications screening program (DiScRi). The study was approved by the local ethics committee. No exclusion criteria were applied, with the exception of excluding records where the time series contained more than 5% ectopic beats.

### Procedures and instrumentation

Participants were rested for a minimum of 5 min, in a supine position on an examination plinth before measuring ABPI and recording heart rate. Examination rooms were all of a comfortable temperature with minimal noise during the measurements. Using a pressure cuff, sphygmomanometer and a Doppler ultrasound unit (Hadeco ES-1000 SPII, Hayashi Denki Co., Tokyo Japan) with an 8 MHz probe, systolic blood pressure (SBP) measurements were obtained from each brachial artery and each posterior tibial artery. The ABPI was calculated by dividing the ankle blood pressure readings by the brachial blood pressure readings to provide a normalized ratio. Mean ABPI scores for each participant were calculated using the left and right foot ABPI results (Brooks et al., 2001).

ECG recordings were made under standardized conditions between 10 a.m. and 3 p.m. using a Powerlab recording system (ADInstruments) and Chart™ software package (version 6). The sampling frequency was set at 1000 Hz, as recommended in the literature (Task Force, 1996), and a 45 Hz low-pass filter was used to eliminate high frequency noise. Three disposable snap ECG electrodes were placed onto the participant's left hip bone, left and right collar bones for a Lead II recoding configuration, as this provided the best indication of the R peaks within the ECG. All ECG signals went through a detailed manual editing process. HRV analyses were performed on the 5-min ECG time series using Kubios HRV analysis software (Tarvainen et al., 2014b).

Time domain analyses quantify the amount of variability in heart rate time series derived from ECG recordings (Chandra et al., 2003). The standard deviation of normal interbeat intervals (SDNN), percentage of successive interbeat intervals differing more than 50 ms (pNN50), the root mean square of the standard deviation (RMSSD), and Poincaré Plot analysis, were chosen as the time-domain indices. Frequency domain analysis quantifies the underlying rhythms in an ECG signal. It partitions the total variance of the heart rate into variance accounted for by the underlying groups of frequencies: low frequency (LF power: 0.04–0.15), which is representative of parasympathetic and sympathetic influence, and high frequency (HF power: 0.15–0.4 Hz), representing parasympathetic influence. Total power as well as the LF/HF ratio were also determined (Lombardi et al., 1996). LFnu and HFnu are the LF power and HF power normalized by total power (Task Force, 1996). The nonlinear measures included detrended fluctuation analysis (DFA), correlation dimension (D2), and sample entropy (SampEn). Detrended fluctuations analysis (DFA) quantifies the presence or absence of complex correlation properties of the R–R intervals (Mäkikallio et al., 2002). DFA α is the gradient of a spectrum of data and can be divided into DFA α_1_, which is an index for short-term (4–11 beats) correlations and DFA α_2_, which is an index for longer (12–64 beats) correlations in an R-R interval time series and outlined in detail at www.physionet.org (Peng et al., 1995).

Sample entropy (SampEn) quantifies the regularity and complexity of time series data such as heart rate (Richman and Moorman, 2000). Large values of SampEn indicate high irregularity, while smaller values of SampEn suggest a more regular signal. Sample entropy (*m, r, N*) is an extension of the ApEn approach developed by Grassberger and others (Grassberger, 1988) and is defined similarly to ApEn. SampEn differs to ApEn in that in SampEn self-comparisons of embedding vectors (RR sequences of *m* = 2 or *m* = 3 beats) are excluded, whereas in ApEn every embedding vector is compared also with itself. Second, SampEn takes the logarithm of the sum of the probabilities rather than the log of each probability. Third, SampEn is more robust for smaller data sets (Richman and Moorman, 2000; Yentes et al., 2013).

## Statistics

A Kruskal-Wallis test followed by a post hoc Mann-Whitney *U*-test were applied to the data following a test for homogeneity (kurtosis > 2). Chi-square analysis was performed to determine group differences for medication use. All data were analyzed using SPSS Version 22 (IBM Pty). Statistical significance was identified using *p* < 0.05.

## Results

ABPI, HRV and demographic data, as well as additional clinical data, was collected for 234 patients attending the diabetes complications screening clinic at Charles Sturt University in Albury, Australia. Using the ABPI range, the cut-off values for the lowest and highest tertile were determined to be <1.07 and >1.23 and were used to divide the participants into normal (*n* = 101), low (*n* = 67) and high (*n* = 66) ABPI groups. Group demographics are shown in Table 1.

**Table 1:** **Physiological differences in the cohort between those with normal, low and high ABPI**.

	**Normal ABPI *n* = 101**	**Low ABPI *n* = 67**	**High ABPI *n* = 66**
Gender (F/M)	57/44	39/28	33/33
Age (years)	61.4 ± 13.3	63 ± 12	59 ± 11
Diabetes Type 2 (years)	4.7 ± 8	4.5 ± 6	2.26 ± 5^%^
Diabetes Type 2 (%)	18.2	13.1	8.9
CVD (years)	0.6 ± 2.8	1.5 ± 4	0.4 ± 2
HT (years)	3.4 ± 8.9	8.1 ± 12	3.2 ± 8^*^^#^
WC (cm)	93.53.4 ± 15.5	100.1 ± 18.4	96.1 ± 12.7
BMI	28.7 ± 6.1	29.7 ± 6.6	28.3 ± 5.5
SBP (mmHg)	133.8 ± 17.4	139.8 ± 18.6^#^	124.5 ± 14^*^^%^
DBP (mmHg)	79.6 ± 8.6	80 ± 10.1	76 ± 9^*^^%^
Average ABPI	1.16 ± 0.05	0.96 ± 0.1^#^	1.31 ± 0.05^*^^%^

* p < 0.05 significant for low vs. high ABPI.

# p < 0.05 normal vs. low ABPI.

% p < 0.05 normal vs. high ABPI; CVD, cardiovascular disease reported; HT, hypertension reported; WC,waist circumference; BMI, body-mass-index; SBP, systolic blood pressure; DBP, diastolic blood pressure; ABPI, ankle-brachial-pressure-index. (Values in mean ± standard deviation).

There were no statistical significant differences between genders in any of the three groups. The group with lower ABPI were slightly older, with a mean age of 63 years. However this difference was not significant. The low ABPI group also had a longer history of DM, with an average duration of 4.5 years, and longer duration of hypertension (*p* < 0.05). However the normal ABPI group had the highest percentage of individuals with diabetes type 2. Differences for systolic blood pressure (SBP) and ABPI were significant between the three groups (*p* < 0.05).

Traditional biomarker results are presented in Table 2. These indicate that only HbA1c was significantly higher in the low ABPI group (Table 2). Cholesterol markers were within normal limits but screening blood glucose levels were slightly elevated in all three groups but not significantly different.

**Table 2:** **Differences in measured blood chemistry between those with normal, low and high ABPI**.

	**Normal ABPI *n* = 101**	**Low ABPI *n* = 67**	**High ABPI *n* = 66**
BGL (mmol/L)	6.81 ± 2.9	6.2 ± 1.9	6.1 ± 2.9
HbA1c (%)	6.5 ± 1.1	6.7 ± 1.2	6.1 ± 0.8^*^^%^
TC (mmol/L)	5.02 ± 1	4.89 ± 1.05	5.1 ± 1.4
Trigs (mmol/L)	1.41 ± 0.9	1.46 ± 0.9	1.2 ± 0.7
HDL (mmol/L)	1.49 ± 0.5	1.51 ± 0.5	1.53 ± 0.5
LDL (mmol/L)	2.89 ± 0.9	2.7 ± 1	3 ± 1.2
TC/HDL	3.6 ± 1	3.5 ± 1.1	3.6 ± 1.3

* p < 0.05 is significant for low vs. high ABPI.

^#^p < 0.05 normal vs. low ABPI.

% p < 0.05 normal vs. high ABPI; BGL, blood glucose level; HbA1c, glycated hemoglobin; TC, total cholesterol; Trigs, triglycerides; HDL, high density lipoprotein; LDL, low density lipoprotein; TC/HDL, total cholesterol: high density lipoprotein ratio. (Values in mean ± standard deviation).

**Figure 2:**
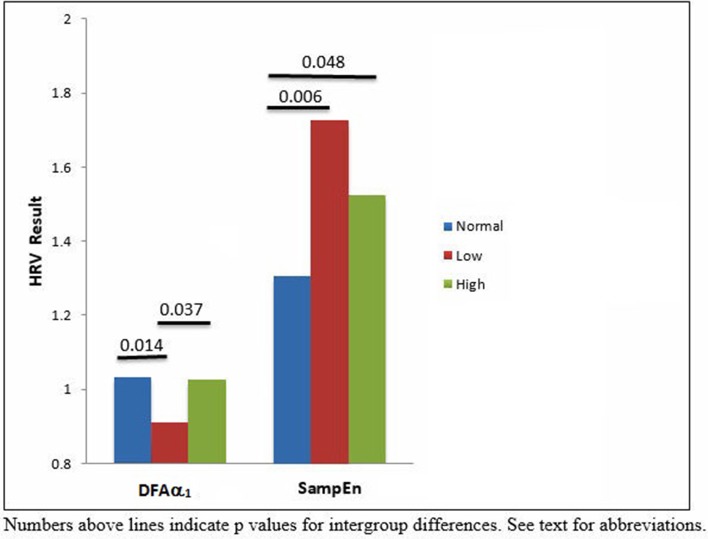
**Nonlinear HRV for normal, low and high APBI centile groups**.

HRV results were divided into time domain, frequency domain and non-linear parameters. The linear measures of LF/HF ratio and LFnu were significantly decreased in the low ABPI compared to the normal ABPI group. Normalized units of low frequency power (LFnu) was lower in both the low ABPI group as compared to the normal ABPI group, indicating a possible reduced sympathovagal balance affecting the low ABPI group more than the high ABPI group. Only the non-linear parameters provided significant differences between the low and high ABPI groups, as shown in Table 3 and Figure 2.

**Table 3:** **Differences in measured HRV between those with normal, low and high ABPI**.

	**Normal ABPI**	**Low ABPI**	**High ABPI**
SDNN (ms)	40.7 ± 19	42.9 ± 18.5	39.6 ± 16.6
RMSSD (ms)	28.1 ± 26.8	31.5 ± 25.3	28.4 ± 19.2
pNN(50) (ms)	11.8 ± 18.3	9.9 ± 13.7	10.4 ± 19.1
LF (ms^2^)	432.9 ± 573.8	432 ± 519.5	430.5 ± 454.7
HF (ms^2^)	385.4 ± 819.2	419 ± 630.7	413.6 ± 673.3
LFnu	57.4 ± 18	50.9 ± 17.3^#^	53.6 ± 19.3
HFnu	41.5 ± 45.1	43 ± 16.3	41.7 ± 18.4
LF/HF	2.3 ± 2.2	1.6 ± 1.7^#^	2.1 ± 2.4
SD1	28.8 ± 25.7	28.7 ± 24.6	22.4 ± 21.4
SD2	56.7 ± 4.42	54.2 ± 21.1	49.6 ± 25.7
DFAα_1_	1.03 ± 0.3	0.91 ± 0.28^#^	1.02 ± 0.3^*^
SampEn	1.3 ± 0.3	1.72 ± 0.6	1.52 ± 0.3^*^^%^

* p < 0.05 is significant for low vs. high ABPI.

# p <0.05 normal vs. low ABPI.

% p < 0.05 normal vs. high ABPI; LF, low frequency power; HF, high frequency power; LFnu, low frequency normalized units; HFnu, high frequency normalized units; LF/HF, ratio of low frequency to high frequency power; DFAα1, short-term scaling for detrended fluctuation analysis; SampEn, sample entropy. (Values in mean ± standard deviation).

**Figure 3:**
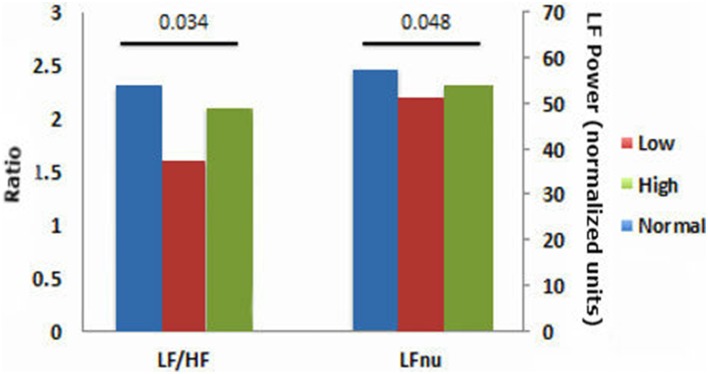
**Time and frequency domain HRV results comparing normal, low and high APBI groups**.

DFAα_1_ was significantly lower in the low ABPI group compared to normal and high ABPI group. SampEn results were significantly higher in the high ABPI as compared to the normal group (Figure 3).

This result is also reflected in the low frequency to high frequency ratio, which is lowest in the low ABPI group. However, both LFnu and the LF/HF ratios were not significantly different between the low and high ABPI group. None of the linear HRV measures indicated a significant difference between the normal and high ABPI group.

Figure 3 emphasizes that only the nonlinear parameters were significantly different between the normal and high ABPI group as well as between the low and high ABPI group.

## Discussion

Network physiology provides a model for investigating complex dynamic processes and interactions between physiological systems that may lead to transitions and new steady states, and enables a clearer understanding of pathophysiology. Self-organizing systems emerging through phase transitions far from equilibrium, have been discussed for various biological systems (Horsthemke and Lefever, 1984; Erdi and Barna, 1985).

Complex pathological metabolic processes are a natural input that drive activity-dependent, self-organizing dynamic networks, such as the peripheral vascular system and cardiovascular system, toward transitions which lead to new, stable albeit pathological forms.

The current work investigated the relationship between cardiac autonomic neuropathy and PVD, in a clinical population attending a diabetes complications clinic. Previous studies have indicated a relationship between CVD and PVD, where PVD was defined by a low ABPI (typically <0.9) (Rose, 2000; Brooks et al., 2001; McDermott McGrae et al., 2003b). However, investigations into the relationship between high ABPI and CVD, and whether there is a different mechanism involved in the coupling between arterial stenosis and CVD vs. vascular calcification and CVD, have not been reported.

Peripheral vascular disease and ABPI are correlated with HRV in people with cardiovascular disease, where HRV and ABPI values are both reduced (Goernig et al., 2008; Ahn and Kong, 2011). Increased HRV, however, has also been suggested to be associated with cardiovascular pathophysiology and this association was further investigated in the current research. The current results show that DFAα_1_ was decreased in the low ABPI group, as compared to the normal ABPI group, indicting a loss of complexity in HRV associated with arterial stenosis. The loss of complexity is possibly associated with an overall change in sympathovagal balance and the significant decrease observed in LFnu power. DFAα_1_, although shown as being significantly different between low and high ABPI, is similar to the DFAα_1_ values shown for the normal ABPI group. Thus the pathophysiology of vascular calcification in our group may not directly affect HRV complexity. Entropy or disorder, however was significantly increased in the high ABPI compared to normal ABBPI group when measured as SampEn. Both reduced or increased HRV may thus be pathological and indicate different PVD pathology (Stein et al., 2005) associated with arterial stenosis and vascular calcification. The extreme but nonsignificant high value of SampEn seen for the low ABPI compared to the normal ABPI group (Table 3) may be due to the current data distribution or HRV results associated with short-term recordings. However the high SampEn may indeed reflect an increased disorder or a difference in regularity of the two data sets (Mayer et al., 2014) and is in line with the decreased complexity and decreased LFnu observed in the low ABPI group. In a previous study of ours we report significant decreased linear HRV being associated with cardiac autonomic neuropathy and associated with a significant increase in nonlinear HRV measures (Tarvainen et al., 2014a). Nonlinear measures of heart rate variability have been shown to be more sensitive in describing the fractal-like, nonlinearity characteristics of the cardiovascular system (Voss et al., 2009). The current results extend the finding that reduced and elevated ABPI correlate with significant changes in autonomic nervous system modulation of the heart (Vowden and Vowden, 2001; Rabkin et al., 2012). A correlation between ABPI and nonlinear HRV measures has also been shown (Jelinek et al., 2013). The current study reports a significant difference in nonlinear DFAα_1_, and SampEn parameters between the low and high ABPI group. Our current findings demonstrate that the relationship between heart rate variability, as an indicator of cardiac rhythm, and ABPI depends on whether PVD is defined as arterial stenosis (low ABPI) or vascular calcification (high ABPI). Additionally, our results show that HRV is significantly different between the arterial stenosis group and the vascular calcification group. This finding suggests that the pathophysiological state of the peripheral vascular and cardiac networks is driven by complex interactions including metabolic processes such as oxidative stress and inflammation. This pathological oxidative stress and/or inflammatory response, leading to either arterial stenosis or vascular calcification also leads to different transitions observed in the heart rate modulated by the ANS. In our data, the development of arterial stenosis and a decreased ABPI, or vascular calcification and elevated ABPI values indicates a possible coupling of the heart rhythm with the state of the peripheral vasculature.

The cut-off value for ABPI, indicating the presence of arterial stenosis, typically range from 0.8 to 1.1 (Hiatt et al., 1995; Moffatt and O'Hare, 1995; Rose, 2000; Brooks et al., 2001; Vowden and Vowden, 2001; McDermott McGrae et al., 2003b). An ABPI of less than 0.90 has been reported to be 95% sensitive and between 50 and 99% specific for angiographic significant arterial stenosis (Applegate, 1993; Olin, 1998). Calcification, however, has been shown to be associated with an ABPI greater than 1.2, although some authors have suggested 1.3 or 1.4 as a cut-off threshold (Jelinek and Austin, 2006; Jelinek et al., 2013; Nashar and Egan, 2014). Both arterial stenosis and non-compressible vascular disease have been previously reported to be associated with higher CVD risk (Di Carli et al., 1999; Lise et al., 2006; Belch et al., 2008; Pop-Busui et al., 2010; Mels et al., 2014).

The clinical use of HRV and ABPI as quantitative measures assumes not only that these tests assessing diabetic neuropathy and hemodynamic function respectively, are correlated, but also that the pathophysiology has either certain common or disparate features. The autonomic nervous system modulates heart rate and also vessel patency. Hence autonomic neuropathy affects the regulation of both blood flow and heart rhythm (Thomas, 2011). Additionally, pathophysiological processes including inflammation and oxidative stress, which affect the autonomic nervous system and endothelial cell function, play a role in disease progression and presentation (Al-Aubaidy and Jelinek, 2014; Laitinen et al., 2014). We investigated whether HRV was significantly different when ABPI was reduced or elevated outside the normal range. Our findings indicate that the interaction between cardiac autonomic neuropathy and vascular dysfunction is multifactorial and differs depending on the pathophysiology associated with PVD.

Network physiology provides a model for understanding complex, nonlinear dynamics associated with the development of discrete vascular and cardiac pathologies as self-organizing networks, which are linked by various functional coupling and feedback interactions through metabolic and neural networks (Bashan et al., 2012). Here we propose that possible pathological changes reported previously manifest as changes in the redox balance of the network. The change in the redox milieu can lead to perturbations in the neural and vessel subsystems/networks and to transitions in function as new steady-state topologies of the networks. Here we describe the transition from a balanced multicomponent network (vascular and cardiac) within a complex metabolic domain to new alternative topologies of these networks. Hence arterial stenosis (low ABPI) and vascular calcification (high ABPI) and become associated with low and high HRV respectively. Low and high HRV values indicate pathology and an increased risk of adverse cardiac events (Stein et al., 2005). Our current findings however indicate that linear and nonlinear HRV measures are sensitive to different characteristics of the RR time series. The possible coupling observed for the vascular and cardiac multistable states may be due to changes in the functional coupling and bidirectional feedback mechanisms associated with the vascular and cardiac networks (Bartsch et al., 2012; Kelso, 2012).

The emphasis of this paper is to describe a novel interaction between subnetworks that manifest as different clinical pathologies and to propose a model based on network physiology, which describes the transitions in these networks. Future work will analyze continuous data from normal, low and high ABPI individuals utilizing Doppler ultrasound technology and will investigate the coupling with HRV, determined from ECG recordings of 30–120 min duration, in order to enhance our understanding of the coupling mechanisms.

## Conclusion

Presence of CVD risk based on HRV showed an association with nonlinear HRV measures, which differentiated between the low and high ABPI group. This suggests that a link between both arterial stenosis or vascular calcification, and heart disease, does indeed exist. These findings may be described by a network physiology model that is based on phase transitions occurring due to system perturbations, coupling strengths and feedback mechanisms that drive dynamic processes to new and different steady states. In addition to shedding light on the complex etiology of PVD through a network physiology framework, the results obtained in this study can be used toward establishing more sensitive diagnostic assessment measures as related to cardiovascular health and functionality.

## Author contributions

HJ conceived and designed the study and obtained the data with HA, DC, and MT did the analysis of the HRV, HA, HJ, KK, and CR analyzed demographic and clinical variables. Interpretation of data and drafting the work as well as revising it critically for important intellectual content was undertaken by all authors. All authors approved of the final version to be published and agreed to be accountable for all aspects of the work in ensuring that questions related to the accuracy or integrity of any part of the work are appropriately investigated and resolved.

### Conflict of interest statement

The authors declare that the research was conducted in the absence of any commercial or financial relationships that could be construed as a potential conflict of interest.
